# Reports of Symptoms Associated with Supraventricular Arrhythmias as a Serious Adverse Drug Reaction in the Spanish Pharmacovigilance Database

**DOI:** 10.3390/ph16081161

**Published:** 2023-08-15

**Authors:** Javier Pueyo-Val, Ana Avedillo-Salas, Pablo Berdún-Viñegra, Olga María Pueyo-Val, Ana Fanlo-Villacampa, Cristina Navarro-Pemán, Francisco Javier Lanuza-Giménez, Ignatios Ioakeim-Skoufa, Jorge Vicente-Romero

**Affiliations:** 1 Department of Pharmacology, Physiology and Legal and Forensic Medicine, Faculty of Medicine, University of Zaragoza, ES-50009 Zaragoza, Spain; 2 Anesthesia and Resuscitation Department, Cruces University Hospital, ES-48903 Barakaldo, Spain; 3 Centro de Salud Báguena, Báguena, ES-44320 Teruel, Spain; 4 Aragon Pharmacovigilance Center, ES-50017 Zaragoza, Spain; 5 WHO Collaborating Centre for Drug Statistics Methodology, Department of Drug Statistics, Division of Health Data and Digitalisation, Norwegian Institute of Public Health, NO-0213 Oslo, Norway; 6 EpiChron Research Group, Aragon Health Research Institute (IIS Aragón), Miguel Servet University Hospital, ES-50009 Zaragoza, Spain; 7 Research Network on Chronicity, Primary Care, and Health Promotion (RICAPPS), Institute of Health Carlos III (ISCIII), ES-28029 Madrid, Spain; 8 Drug Utilisation Work Group, Spanish Society of Family and Community Medicine (semFYC), ES-08009 Barcelona, Spain

**Keywords:** pharmacovigilance, adverse drug reaction reporting systems, drug-related side effects and adverse reactions, cardiovascular system, supraventricular arrhythmias, atrial fibrillation, sinus dysfunction, sinus bradycardia, patient safety

## Abstract

This study aimed to determine the type of drugs reported as suspected of causing severe supraventricular arrhythmias from the Spanish Human Pharmacovigilance System database. A total of 1053 reports were analysed, of which 526 (50%) were on men and 516 (49%) were on women. The most affected age group was the over-65s, with 593 reports (56%). Of the 1613 drugs, those belonging to the cardiovascular system (ATC Group C) were the most numerous (414 reports, 26%), with digoxin being the most frequent drug (49 reports, 12%). Other common groups were antiinfectives for systemic use (ATC Group J; 306 reports, 19%), antineoplastic and immunomodulating agents (ATC Group L; 198 reports, 12%), and nervous system drugs (ATC Group N; 185 reports, 11%). The most common supraventricular arrhythmia was atrial fibrillation (561 reports, 51%). Regarding outcomes, 730 (66%) patients recovered, 76 (7%) did not recover, 25 (3%) recovered but with sequelae, and 23 (2%) resulted in death. This study revealed that certain drugs have reported to be associated more frequently to supraventricular arrhythmias as serious adverse reactions, especially in the older population. Proper clinical management and effective strategies to ensure medication appropriateness should always be considered to improve patient safety when prescribing drugs.

## 1. Introduction

Cardiovascular diseases are one of the leading causes of morbidity and mortality worldwide. Amongst the multiple aetio-pathophysiologic factors, certain medicines may have a significant contribution; it has been estimated that drugs are amongst the most common causes of morbidity and mortality [1,2,3,4,5]. Arrhythmias are classified as bradyarrhythmias (slow rhythm, less than 60 beats per minute) and tachyarrhythmias (more than 100 beats per minute), which are subdivided into supraventricular and ventricular according to its origin. Supraventricular arrhythmias are a heterogeneous group of electrical rhythm disturbances in the heart that originate above the ventricles. Traditionally, supraventricular arrhythmias have been considered less harmful than those of ventricular origin. However, the sudden presence of a fast or slow rhythm of supraventricular origin can be just as harmful, and even fatal, as ventricular tachyarrhythmias. Supraventricular arrhythmias include clinical and electrocardiographic changes from sinus tachycardia to supraventricular tachycardia, including atrial flutter and the most prevalent arrhythmia in the population—atrial fibrillation. On the other hand, bradyarrhythmias include various conditions such as sinus bradycardia and atrioventricular block [6,7].

Drug-induced supraventricular arrhythmias include cardiovascular drugs, alcohol, stimulants, anticancer agents, and immunomodulators [8,9,10,11,12,13]. The mechanisms by which drug-induced supraventricular arrhythmias occur may vary from drug to drug. Thus, some stimulants act via catecholaminergic augmentation, while adenosine shortens atrial effective refractory period and promotes pulmonary vein ectopy [13]. Supraventricular arrhythmias may result in serious or fatal outcomes and are frequently listed as adverse drug reactions (ADRs) in the medication package insert (for example, Summary of Product Characteristics, SmPC, and Prescribing Information, PI) of several drugs. However, this field is poorly studied, and more research is needed, considering the great challenges adverse drug reactions pose to healthcare professionals and public health systems worldwide.

Spontaneous reporting of suspected ADRs can create an agile and rapid system for identifying potential ADRs that have not yet been identified, known in pharmacovigilance as “signal generation”. This is of particular importance, especially in an ageing population, where the prevalence of multimorbidity (co-existence of two or more chronic conditions) and polypharmacy (the concomitant use of multiple medications) is continuously increasing, resulting in a higher risk of additional morbidity, potentially inappropriate medication, and drug-related adverse events [14,15,16,17,18,19,20]. The “signals” of potential drug-related side effects and adverse reactions can be further studied in depth by regulatory authorities, as well as the participation of notifiers [21,22]. If a possible association with the medicine is confirmed, all necessary updates will be then considered for inclusion in the medication package insert and other relevant actions may be required, as the publication of specific assessment reports [23]. The WHO Programme for International Drug Monitoring was created in 1968 to ensure that evidence about harm to patients was collected from as many sources as possible. This enables individual countries to be alerted to patterns of harm emerging across the world, but which might not be evident from their local data alone. With more than 170 full members and associate members in 2022, the programme covers about 99% of the world’s population [24]. This study aimed to identify and describe reports of severe supraventricular arrhythmias, as possible adverse drug reactions, based on spontaneous reports of suspected adverse drug reactions submitted to the Spanish Human Pharmacovigilance System Database (FEDRA^®^) [23].

## 2. Results

### 2.1. General Data on Reports

**Figure 1:**
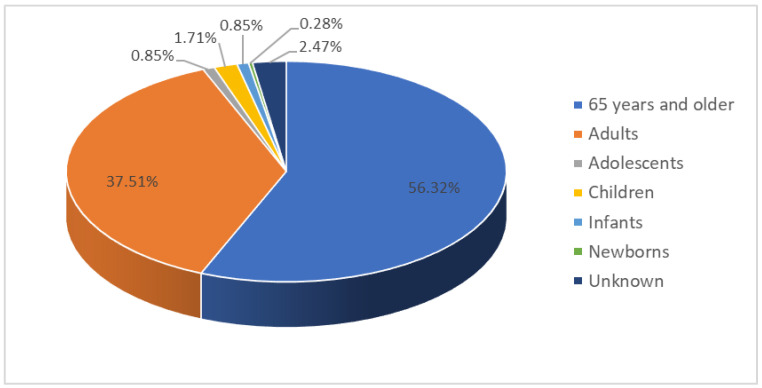
Age distribution of the patients in the spontaneous reports of suspected adverse drug reactions that resulted in supraventricular arrhythmias.

There were 1053 reports of supraventricular arrhythmias during the study period; 526 reports (50%) on male patients, 516 (49%) on females, and in 11 reports (1%), there was no information regarding sex. The mean age was 64.59 years with a standard deviation of 19.97 years, with ages ranging from 1 day to 96 years. By age group, 593 reports corresponded to patients older than 65 years (56.32%), 395 to adults (37.51%), nine to adolescents (0.85%), 18 to paediatric population (1.71%) between one and 13 years old, nine to infants (0.85%), and three to newborns (0.28%); there was no information regarding age in 26 reports (2.47%), as shown in Figure 1.

In the analysis of the age group 65 years and older, 311 patients (52%) were between 76 and 89 years, 238 (40%) between 65 and 75 years, and 33 (6%) over 90 years old; in 11 reports (2%), the exact age was unknown.

According to the profile of the notifier, 677 reports (61%) were submitted by physicians, 298 (27%) by pharmacists, 50 (5%) by non-healthcare professionals, 58 (5%) by the user himself, and 11 (1%) by nursing professionals; in eight reports (1%), the profession of the notifier was not specified.

Concerning the workplace profile from which the professional reports were made, 638 reports (57.8%) were generated by hospitals, 323 (29.3%) non-hospital centres, and 142 (12.9%) of the reports did not specify the workplace profile. Of the latter, 76 (53.52%) were completed by medical professionals, 35 (24.65%) by the user, 18 (12.68%) by unspecified health professionals, nine (6.34%) by pharmacists, one (0.7%) by nurses, and three (2.11%) were unspecified.

### 2.2. Arrhythmias Adverse Drug Reactions

In a total of 1053 reports, 1613 suspected drugs were recorded. The most common drug group was the ATC Group C, corresponding to drugs for the cardiovascular system, with 414 registrations (25.67%). Other ATC groups with a high prevalence of spontaneous reports of suspected drug-induced supraventricular arrhythmias were antiinfectives for systemic use (Group J) with 306 registrations (18.97%), antineoplastic and immunomodulating agents (Group L) 198 (12.28%), and drugs for the nervous system (Group N) with 185 reports (11.47%).

**Figure 2:**
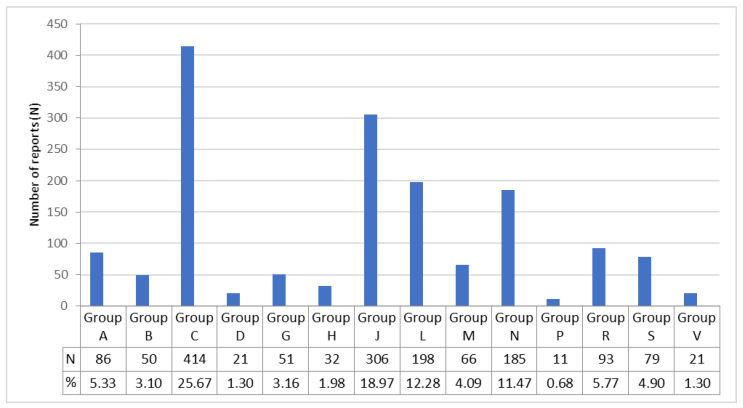
Drugs included in the spontaneous reports of suspected adverse drug reactions that resulted in supraventricular arrhythmias. Drugs are classified according to the World Health Organisation’s Anatomical, Therapeutic, Chemical (ATC) classification system. The main anatomical groups refer to: A, alimentary tract and metabolism; B, blood and blood forming organs; C, cardiovascular system; D, dermatologicals; G, genito urinary system and sex hormones; H, systemic hormonal preparations, excl. sex hormones and insulins; J, antiinfectives for systemic use; L, antineoplastic and immunomodulating agents; M, musculo-skeletal system; N, nervous system; P, antiparasitic products, insecticides and repellents; R, respiratory system; S, sensory organs; and V, various.

The remaining ATC Groups were distributed as follows: 93 Group R (respiratory system), 86 Group A (alimentary tract and metabolism), 79 Group S (sensory organs), 66 Group M (musculo-skeletal system), 51 Group G (genito urinary system and sex hormones), 50 Group B (blood and blood forming organs), 32 Group H (systemic hormonal preparations, excluding sex hormones and insulins), 21 Group D (dermatologicals), 21 Group V (various) and 11 Group P (antiparasitic products, insecticides and repellents), as shown in Figure 2.

Within the ATC Group C, 45% of the reports included only the following five drugs: digoxin, bisoprolol, amiodarone, carvedilol and diltiazem, as shown in Table 1.

In ATC Group J (antiinfectives for systemic use), 183 (59.8%) correspond to reports included as vaccines for the treatment of COVID-19 (ATC J07BX03), with an overall percentage among all drugs of 11.3%. The rest of the drugs in the group present a very heterogeneous distribution except moxifloxacin with 13 reports (ATC J01MA14), and azithromycin with eight (ATC J01FA10), representing 0.8% and 0.5%, respectively.

In ATC Group L, corresponding to antineoplastic and immunomodulating agents, only ibrutinib (ATC L01XE27), a direct protein kinase inhibitor, stands out in 21 reports (1.3%).

Within ATC Group N (nervous system), we obtained 185 cases. Among them, the subgroup of psychoanaleptics (ATC N06), with 66 cases (35.67%), is the most numerous, and within it, antidepressants (ATC N06A) with 43 cases. Drugs belonging to the subgroup ATC N05 (psycholeptics) were collected with 30 cases (16.21%), and antipsychotics (ATC N05A), with 20 cases (10.81%), accounted for the highest number of cases.

The analysis of the drugs in the remaining ATC Groups shows a very uneven distribution, with few reports for each drug. Only salbutamol (ATC R03AC02) stands out with 21 reports (1.3%) (Supplementary Table S1).

**Figure 3:**
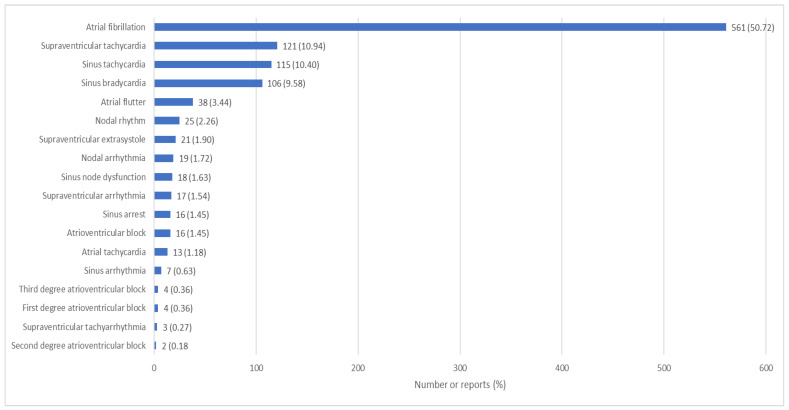
Supraventricular arrhythmias reported in FEDRA^®^ according to the preferred term of the MedDRA^®^ classification.

Regarding the reported arrhythmias, 561 (50.72%) were atrial fibrillation, 121 (10.94%) supraventricular tachycardia, 115 (10.40%) sinus tachycardia, 106 (9.58%) sinus bradycardia, 38 (3.44%) atrial flutter, 25 (2.26%) nodal rhythm, 21 (1.90%) supraventricular extrasystole, 19 (1.72%) nodal arrhythmia, 18 (1.63%) sinus dysfunction, 17 (1.54%) supraventricular arrhythmia, 16 (1.45%) AV block, 16 (1.45%) sinus arrest, 13 (1.18%) atrial tachycardia, 7 (0.63%) sinus arrhythmia, 4 (0.36) third degree AV block, 4 (0.36) first degree AV block, 3 (0.27) supraventricular tachyarrhythmia and 2 (0.18) second degree AV block, as shown in Figure 3 (detailed information is given in Supplementary Table S2).

Out of the 1053 reports of the study sample, 29 (2.75%) were coded each with more than 1 PT.

Most of the ADRs analysed (99%) followed a compatible time sequence, while 1% had a compatible but inconsistent time sequence, or more information needed to be collected.

Regarding the clinical outcome of the suspected adverse reaction, 680 cases (65%) recovered, 99 (13%) were recovering, 76 (7%) did not recover, 28 (3%) recovered with sequelae, and in 23 cases (2%), death occurred; in 138 cases (13%), no information on clinical outcome was recorded. Out of the 23 cases with a fatal outcome, 56.53% were female and 39.13% male. Regarding age, 86.9% of these reports belong to the 65 years and older age group (Supplementary Materials Table S3). Data regarding drug withdrawal and clinical outcome showed that in 1393 cases (73.08%), the drug was withdrawn, and the patient improved. In 48 (2.52%), the drug was withdrawn but the reaction did not improve. In 50 (2.62%) of them, the patient improved by keeping the drug but with timely treatment for the reaction caused, and in 20 (1.05%), the drug was not withdrawn and did not improve. Also, in 70 cases (3.67%), the clinical evolution was fatal or irreversible. In 325 cases (17.05%), no information was collected in this regard.

Regarding prior knowledge of a possible adverse reaction of the reported drug used, in 1193 reports (62.59%), this possibility was well known, and in 574 (30.11%), it was unknown. A total of 131 reports (6.87%) had occasional references in the literature, and eight cases (0.42%) were against the drug-reaction relationship.

Of the reported reactions where the reaction was well known, the drug was withdrawn in 914 (76.6%), and the patient improved. In only 15 reports (1.26%), the drug was withdrawn, and the patient did not improve. From the 574 reports where the ADR was unknown, 340 of the patients (28.5%) improved when the drug was withdrawn, and only 31 (2.6%) did not improve when the drug was withdrawn.

Likewise, the effects of re-exposure to the drug were as follows: 63 (3.3%) of them had a fatal or irreversible outcome, 33 (1.73%) had the same reaction again, in 19 (1%), no adverse reaction was recorded again, five (0.26%) had a reaction with another drug and in 1786 (93.7%), the drug was not reintroduced, or this circumstance was not recorded.

## 3. Discussion

In this study, we analysed spontaneous reports of suspected ADRs of supraventricular tachycardia based on information submitted to a national public pharmacovigilance database. We identified drugs that were implicated in these reports, considering the severity of the suspected adverse reactions, the clinical outcome, and the withdrawal or not of the drug. Data analysis showed that most of the drugs belonged to the cardiovascular group (ATC Group C); amongst them, digoxin and beta-blockers were the most common.

Our study showed no significant sex differences in the occurrence of supraventricular arrhythmias as a suspected adverse drug reaction. However, most studies reported a slight increase in notifications of ADRs in women, especially in the older population, and it is more significant as age increases [15,25]. As shown in previous studies, in the oldest old population, the female sex is more prevalent than the male sex, especially in the nonagenarian and centenarian populations, with significant differences in the clinical profile and the use of medications [26,27]. It is well known that most people aged 65 years and older live with multiple chronic conditions and polypharmacy and a high risk of inappropriate prescriptions, drug–drug and drug–disease interactions, ADRs and related negative outcomes [28,29,30]. This is important, considering that this age group is underrepresented in clinical trials. The prevalence of arrhythmias in the older population is rising, and various factors could be associated with its increase, for example, comorbidity and concomitant prescriptions [26,27,28,29], age-related changes in pharmacodynamics and pharmacokinetics [31], cardiophysiologic changes, for example, increased myocyte (reflected as hypertrophy) and calcification of cardiac conduction tissue [32], etc.

In our study, more than half of the adverse reactions were reported in the over-65 age group, and almost half were in the 65–75 age group. This could be partly attributed to a higher mean number of medications per individual in the older population compared to younger adults [1,15,25], and considering that most individuals 65 years and older belong to the age group 65–74 years old [14]. The second most prevalent age group, with approximately one-third of the submitted reports, was the working-age adults aged 18–64 years old. Considering that the reports collected and analysed for this study were classified as serious, the cost of the adverse reaction could be added to the cost of temporary incapacity for work if the patient was employed. An important finding was that spontaneous reports of suspected adverse drug reactions that resulted in supraventricular arrhythmias were generated for patients younger than 18, although the occurrence was much lower. Considering the low representation of this age group in clinical trials, it is crucial to closely monitor the clinical evolution following a prescription and adequately report any possible adverse drug reactions [33].

Healthcare professionals generated most of the reports, most of whom were physicians. This is not surprising as this adverse drug reaction is a clinical entity that needs a diagnosis by a medical doctor. Complete atrial fibrillation arrhythmia was the most common form of arrhythmias reported in more than half of the cases. This is the most common type of arrhythmia, affecting people of all ages and mostly individuals 65 years and older [34].

Our data showed that most patients recovered to their pre-adverse reaction status, but a small number had some sequelae. Death was even recorded, which is not insignificant given that supraventricular arrhythmias have always been considered to have a certain degree of banality [6,35].

The most common drugs that were implicated in the spontaneous reports of suspected adverse drug reactions that resulted in supra-ventricular arrhythmias were drugs used to treat cardiovascular conditions (ATC Group C). Among them, digoxin and beta-blockers were the most common, which align with the main indication and their own adverse reactions. Beta-blocking agents are amongst the most common drugs in patients with chronic polypharmacy, with an increased use during the last years in patients 65–74 years old [30]. Amiodarone and calcium antagonists also stand out, as arrhythmias are involved in various cardiovascular diseases [4,36,37]. The proarrhythmic effects of antiarrhythmic drugs are well known in the medical literature as being essential to recognize the aetiology (for example, drug-induced origin) of the arrythmias to properly treat it [38].

The second group with the highest rate of reports of severe supraventricular arrhythmias is the group of systemic antiinfectives. It is important to note that this group’s highest percentage of drugs corresponds to reports of intramuscular COVID-19 vaccines, much higher than other antiinfective drugs, such as moxifloxacin and azithromycin [39]; however, it seems unlikely that this observation can be extrapolated to a non-pandemic period [25].

Antineoplastic and immunomodulatory agents represent the third group in notifications. Overall, considering the exceptional nature of the pandemic period, the first (Group C, cardiovascular system drugs) and third (Group L; Antineoplastic and immunomodulating agents) groups are consistent with the distribution of morbidity and mortality both in Spain and worldwide, so it seems logical that the greater the number of treatments for these groups, the more significant the increase in adverse reactions [40,41].

Drugs administered for the nervous system make up the fourth largest group. Despite a very heterogeneous distribution, sedatives and antipsychotics account for the largest share, which seems to align with the increase in these pathologies in recent years, so an increase in adverse reactions is also expected [42].

In the respiratory group (ATC Group R), salbutamol, a β-2 agonist widely used in asthma, was the most common drug that was implicated in the reports of supraventricular arrhythmias. Like others of the cardiovascular group (ATC Group C) that were reported to cause potential adverse reactions, this drug has a well-known association with arrhythmic disorders by stimulating cardiac β-1 receptors [43].

In more than half of the reports, the adverse drug reaction was known, and when the drug was withdrawn, the patient improved in up to 70% of the cases. Only in a small number of the identified cases was it impossible to withdraw the suspected drug and use another one. It is important to highlight that death and irreversible effects were reported as a clinical outcome of the suspected adverse reaction. Reports with a fatal outcome show a higher percentage of female sex. This idea is consistent with the higher mortality from arrhythmias in women due to different reasons [44,45].

These findings are significant from a clinical perspective, considering that, in most of the identified drugs that were implicated in the spontaneous reports of supraventricular arrhythmias, this drug-related adverse reaction is included in the medication package insert. This observation emphasizes the urgent need to design and implement effective strategies to ensure appropriate prescribing in everyday clinical praxis and properly identify and treat adverse drug reactions [46]. Despite preventative strategies described in the literature, there is little point in detecting and predicting ADRs if interventions to counteract them are lacking [47]. It is worthy to mention that an educational program could have a significant impact on knowledge, attitude and practice of healthcare professionals [48].

This work is a large-scale descriptive study with data from a national public pharmacovigilance database. For the identification of drugs, the World Health Organization’s ATC classification was used, as an international gold standard of drug utilization studies, allowing for plausible comparisons between countries [49,50,51]. Similarly, for the identification of the adverse events we used the tool MedDRA^®^ developed by the International Council for Harmonisation of Technical Requirements for Pharmaceuticals for Human Use [52]. Data from the Pharmacovigilance System are useful for identifying associations between drugs and adverse events—especially the not yet known—but not causation, incidence or risk of an adverse reaction [23,53].

Data for this study were extracted from the Spanish Pharmacovigilance Database, and thus, results cannot be generalized to other populations; further studies are needed in different populations. A very common limitation in pharmacovigilance studies is underreporting, especially for well-known adverse drug reactions. Furthermore, the reporting rate may vary depending on the type and severity of the reaction, the drugs involved and whether a new drug is being used. Considering the nature of the present work, certain limitations are present regarding the clinical diagnosis of supraventricular arrhythmias (not all cases, or classification of arrhythmias, were clinically confirmed by a physician); however, most spontaneous reports were generated by healthcare professionals. The spontaneous reports are reporting signals and symptoms associated with supraventricular arrhythmias due to the diagnosis not being provided in each case. Also, it is challenging to distinguish between toxic effects due to the recommended dose and those due to overdose, so this point has not been analysed [1,4]. In addition, atrial fibrillation can present haemodynamic complications and serious situations due to a fast or slow rhythm, so it is not possible to determine exactly whether the collected data are due to very fast rates or, on the contrary, slow rhythms, and thus, it was not possible to conclude exactly this term. Despite such limitations, pharmacovigilance studies can generate “signals” of potential drug-related side effects and adverse reactions that can be further studied to explore causality and discover underlying aetio-pathophysiologic mechanisms.

## 4. Materials and Methods

### 4.1. Study Design and Population

This study is a retrospective analysis of serious spontaneous reports of adverse drug reactions submitted to the Spanish Pharmacovigilance database FEDRA^®^ of the Spanish Pharmacovigilance System for Medicinal Products for Human Use (SEFV-H) [23]. In the present work, we followed a similar methodological approach as followed in previous publications [54]. In this study, we included reports submitted to FEDRA^®^ from 1 January 2000 to 1 June 2022. FEDRA^®^ is a national public database in Spain containing reports of suspected ADRs of drugs for human use and adverse events following vaccination [23]. Reports can be submitted by everyone—healthcare professionals and citizens—to the Autonomous Pharmacovigilance Centres, either directly or through the electronic form available at www.notificaram.es (accessed on 10 July 2023), as well as through pharmaceutical laboratories; cases that have occurred in Spain and are identified through the European Medicines Agency (EMA) review of the scientific bibliography are also included in the database [23]. In Spain, each Autonomous Community has a Pharmacovigilance Centre responsible for registering the notifications in FEDRA^®^. The data are validated and registered according to international standards and pre-established criteria; all data are anonymised. The Pharmacovigilance Centres form the SEFV-H, together with the Spanish Agency of Medicines and Medical Devices (AEMPS), which coordinates the system. The regulatory authorities further investigate the signs of potential risk, and if a possible association with the medicine is confirmed, information is added to the datasheet and package leaflet [23].

Since FEDRA^®^ is a publicly available and anonymized database, and this work does not include any studies with human participants or animals performed by any of the authors, institutional review board approval and informed consent were waived for this study.

### 4.2. Selection of Reports

Serious adverse reactions have been defined as any adverse reaction that “causes death, is likely to be life-threatening, requires hospitalisation or prolongation of existing hospitalisation, results in significant or persistent disability or impairment, or constitutes a congenital anomaly or birth defect. For reporting purposes, suspected adverse reactions that are considered medically significant, even if they do not meet the above criteria, such as those that put the patient at risk or require intervention to prevent them, shall also be treated as serious for any of the above outcomes. Similarly, for reporting purposes, all suspected transmission of an infectious agent via a medicinal product shall be treated as serious” [55].

Clinical manifestations of ADRs were coded using the MedDRA^®^ (Medical Dictionary for Regulatory Activities) terminology [52], which includes the “System Organ Classes (SOC) “Cardiac Disorders”, the “High-Level Group Terms” (HLGT) “Cardiac Arrhythmias”, the “High-Level Terms” (HLT) “Supraventricular Arrhythmias and Cardiac Conduction Disorders” and the preferred terms (PT), that best define the reaction of supraventricular arrhythmias: atrial flutter, nodal arrhythmia, sinus arrhythmia, supraventricular arrhythmia, atrioventricular (AV) block, first degree AV block, second degree AV block, complete AV block, sinus bradycardia, nodal dysfunction, supraventricular extrasystole, atrial fibrillation, sinus arrest, nodal rhythm, atrial tachycardia, supraventricular tachyarrhythmia, sinus tachycardia, and supraventricular tachycardia.

The following variables were available in the reports: sex, age, profile of the reporter, indication leading to drug administration, actions taken in response to the reaction, classification of the active substance reported, outcome, time sequence, alternative causes and prior knowledge of the adverse reaction described for that active substance, as well as the effect of withdrawal and re-exposure of the patient to the drug in question.

The age variable was grouped as follows: newborn, infant (one month to one year), child (one to 13 years), adolescent (14 to 17 years), adult (18 to 64 years) and over 65 years.

The drugs are classified according to the World Health Organisation’s Anatomical, Therapeutic, Chemical (ATC) classification system. In the ATC classification system, the active substances are classified in a hierarchy with five different levels. The system classified drugs into 14 anatomical main groups. Each ATC main group is divided into 2nd levels, which could be either pharmacological or therapeutic groups. The 3rd and 4th levels are chemical, pharmacological, or therapeutic subgroups and the 5th level is the chemical substance [49,50,51].

The outcomes of the serious adverse reaction were categorised as follows: recovered, recovered with sequelae, recovering, not recovered, fatal and unknown.

### 4.3. Statistical Analysis

The frequency distribution of the percentages of each category was calculated for each qualitative variable, and for the quantitative variables, the indicators of central tendency (mean) and dispersion (standard deviation or percentiles) were calculated. The data were analysed using SPSS Statistics for Windows, Version 29 (IBM Corp., Armonk, NY, USA).

## 5. Conclusions

Several drugs were identified to potentially cause supraventricular arrhythmias as a serious adverse reaction based on information extracted from spontaneous reports submitted to a national public pharmacovigilance database. We considered the severity of the suspected adverse reactions, the clinical outcome, and the implicated drug’s withdrawal. Most of the identified drugs were medicines used to treat cardiovascular conditions; amongst them, digoxin and beta-blockers. Our data revealed spontaneous reports of supraventricular arrhythmias may be generated for patients of both sexes and all age groups, mostly in the older population. We observed severe cases and reports where the clinical outcome was fatal. It is essential to design and implement effective strategies to ensure proper clinical management of the patients and appropriate prescribing in all clinical settings to improve patient safety. Spontaneous reporting of ADRs presents several advantages: it is a simple, quick and economical method enabling the generation of hypotheses and identification of new potential safety concerns involving drugs—notably rare, infrequent or unexpected events.

## Figures and Tables

**Table 1:** The most common cardiovascular drugs (Group C of the Anatomical, Therapeutic, Chemical-ATC-classification) implicated in spontaneous reports of suspected drug-related supraventricular arrhythmias.

Drug	ATC	Therapeutic Subgroup	n	%
Digoxin	C01AA05	Cardiotonic glycoside	49	12
Bisoprolol	C07AB07	Selective ß-blocking agents	45	11
Amiodarone	C01BD01	Class III antiarrhythmic	37	9
Carvedilol	C07AG02	α- and ß-blocking agents	29	7
Diltiazem	C08DB01	Benzothiazepine derivatives	26	6

## Data Availability

Data is contained within the article.

## Supplementary Materials

The following supporting information can be downloaded at: https://www.mdpi.com/article/10.3390/ph16081161/s1. Table S1. Prescribe individual drugs most often involved in case reports of symptoms associated with supraventricular as a serious arrhythmias adverse drug reactions in the Spanish Pharmacovigilance Database (n ≥ 5). Table S2. Drugs mainly reported as suspected by Preferred Terms of the MedDRA classification. Table S3. Distribution of reports in FEDRA^®^ according to Preferred Term of the MedDRA classification with a fatal outcome.
