# Supplementary material for: Reports of Symptoms Associated with Supraventricular Arrhythmias as a Serious Adverse Drug Reaction in the Spanish Pharmacovigilance Database
Source: Pharmaceuticals (Basel). 2023 Aug 15;16(8):1161. doi: 10.3390/ph16081161 (PMC10457936; doi:10.3390/ph16081161)
Supplement: Supplementary file 1 [file pharmaceuticals-16-01161-s001.zip › pharmaceuticals-2538984-supplementary.pdf]

Supplementary Materials: Reports of Symptoms Associated with Supraventricular Arrhythmias as a Serious Adverse Drug Reaction in the Spanish Pharmacovigilance Database

Table S1. Prescribe individual drugs most often involved in case reports of symptoms associated with supraventricular as a serious arrhythmias adverse drug reactions in the Spanish Pharmacovigilance Database ($n \geq 5$).

Drug	N	%
Amitriptilin	5	0.3
Carbidopa	5	0.3
Clavulanic acid	5	0.3
Lacosamide	5	0.3
Paclitaxel	5	0.3
Pregabalin	5	0.3
Propofol	5	0.3
Risperidone	5	0.3
Silodosin	5	0.3
Trastuzumab	5	0.3
Covid-19, viral vector, non-replicating (Ad26.COV2-S [recombinante])	5	0.3
Valsartan	5	0.3
Acetylsalicylic acid	6	0.4
Amlodipine	6	0.4
Anagrelide	6	0.4
Dasabuvir	6	0.4
Dutasteride	6	0.4
Infliximab	6	0.4
Levodopa	6	0.4
Lopinavir	6	0.4
Losartan	6	0.4
Metformin	6	0.4
Ombitasvir	6	0.4
Paritaprevir	6	0.4
Quetiapine	6	0.4
Ribavirin	6	0.4
Teriparatide	6	0.4
Tramadol	6	0.4
Ustekinumab	6	0.4
Influenza, inactivated, split virus or surface antigen	6	0.4
Papillomavirus (human types 16, 18)	6	0.4
Bortezomib	7	0.4
Donepezil	7	0.4
Formoterol	7	0.4
Ipratropium bromide	7	0.4
Lenalidomide	7	0.4
Levofloxacin	7	0.4
Levothyroxine	7	0.4
Mirtazapine	7	0.4
Nebivolol	7	0.4
Paracetamol	7	0.4
Potassium	7	0.4

Rituximab	7	0.4
Varenicline	7	0.4
Adalimumab	8	0.5
Alendronic acid	8	0.5
Dexamethasone	8	0.5
Etoricoxib	8	0.5
Timolol	8	0.5
Venlafaxine	8	0.5
Abiraterone	9	0.6
Zoledronic acid	9	0.6
Azithromycin	9	0.6
Furosemide	9	0.6
Hydroxychloroquine	9	0.6
Rivastigmine	9	0.6
Metamizole	10	0.7
Hydrochlorothiazide	11	0.7
Enalapril	12	0.7
Mirabegron	12	0.7
Ritonavir	12	0.7
Spironolactone	13	0.8
Moxifloxacin	13	0.8
Ivabradine	14	0.9
Tamsulosin	14	0.9
Atenolol	15	0.9
Flecainide	15	0.9
Covid-19, RNA-based vaccine (nucleoside modified) / Elasomeran	19	1.2
Verapamil	20	1.2
Salbutamol	21	1.3
Cilostazol	23	1.4
Ibrutinib	24	1.5
Diltiazem	26	1.6
Covid-19, viral vector, non-replicating (ChAdOx1-S [recombinant])	28	1.7
Carvedilol	29	1.8
Amiodarone	37	2.3
Bisoprolol	45	2.8
Digoxin	49	3.0
Covid-19, RNA-based vaccine (nucleoside modified) / Tozinameran	78	4.8

Table S2. Drugs mainly reported as suspected by Preferred Terms of the MedDRA classification.

Preferred terms	N	Drug
Atrial flutter	5	Covid-19, RNA-based vaccine (nucleoside modified) / Tozinameran
Nodal arrhythmia	5	Verapamil
Sinus arrhythmia	1	Atosiban<br>Bisoprolol<br>Cilostazol<br>Eslicarbazepine<br>Ibuprofen<br>Methylphenidate<br>Nebivolol<br>Trazodone
Supraventricular arrhythmia	4	Papillomavirus (human types 16, 18)
Atrio-ventricular (AV) block	41	Clavulanic acid
First degree AV block	1	Carvedilol<br>Cilostazol<br>Desloratadine<br>Mepiramine<br>Pantoprazole
Second degree AV block	1	Moxifloxacin
Complete AV block	4	Diltiazem
Sinus bradycardia	19	Bisoprolol
Nodal dysfunction	0	0
Supraventricular extrasystole	6	Covid-19, RNA-based vaccine (nucleoside modified) / Tozinameran
Atrial fibrillation	68	Covid-19, RNA-based vaccine (nucleoside modified) / Tozinameran
Sinus arrest	3	Diltiazem
Nodal rhythm	5	Amiodarone<br>Bisoprolol<br>Digoxin
Atrial tachycardia	3	Covid-19, RNA-based vaccine (nucleoside modified) / Tozinameran
Supraventricular tachyarrhythmia	1	Covid-19, viral vector, non-replicating (Ad26.COV2-S [recombinante])
Sinus tachycardia	27	Covid-19, RNA-based vaccine (nucleoside modified) / Tozinameran
Supraventricular tachycardia	12	Covid-19, RNA-based vaccine (nucleoside modified) / Tozinameran

Table S3. Distribution of reports in FEDRA® according to Preferred Term of the MedDRA classification with a fatal outcome.

Report	Group AGE	SeX	Drug	Preferred term
**1**	65 years and older	Male	Azithromycin<br>Hydroxychloroquine<br>Lopinavir<br>Ritonavir<br>Tocilizumab	Atrial flutter
**2**	65 years and older	Female	Digoxin	Sinus bradycardia
**3**	65 years and older	Female	Iomeprol	Sinus bradycardia
**4**	65 years and older	Female	Bisoprolol	Sinus bradycardia
**5**	65 years and older	Male	Cisplatin<br>Gemcitabine<br>Paclitaxel	Atrial fibrillation
**6**	Unknown	Unknown	Amiodarone	Atrial fibrillation
**7**	65 years and older	Male	Pregabalin	Atrial fibrillation
**8**	65 years and older	Female	Levosimendan	Atrial fibrillation
**9**	65 years and older	Female	Adalimumab<br>Metrotexate	Atrial fibrillation
**10**	65 years and older	Female	Ertapenem	Atrial fibrillation
**11**	65 years and older	Male	Fibrinogen, human<br>Thrombin	Atrial fibrillation
**12**	65 years and older	Female	Enoxaparin	Atrial fibrillation
**13**	65 years and older	Female	Irinotecan<br>Panitumumab	Atrial fibrillation
**14**	65 years and older	Male	Adalimumab<br>Azathioprine	Atrial fibrillation
**15**	65 years and older	Female	Amiodarone<br>Digoxin	Atrial fibrillation
**16**	Adult	Female	Cytarabine<br>Fludarabine<br>Idarubicin	Atrial fibrillation
**17**	65 years and older	Male	Acetylsalicylic acid<br>Warfarin	Atrial fibrillation
**18**	Adult	Male	Covid-19, RNA-based vaccine (nucleoside modified) / Elasomeran	Atrial fibrillation
**19**	65 years and older	Male	Amphotericin B	Supraventricular tachycardia
**20**	65 years and older	Female	Tolterodine	Supraventricular tachycardia
**21**	65 years and older	Female	Influenza, inactivated, split virus or surface antigen	Supraventricular tachycardia
**22**	65 years and older	Male	Ipratropium bromide<br>Mesna	Supraventricular tachycardia
**23**	65 years and older	Female	Iopromide	Supraventricular tachycardia
